# Innovative surface bio-functionalization by fungal hydrophobins and their engineered variants

**DOI:** 10.3389/fmolb.2022.959166

**Published:** 2022-08-11

**Authors:** Ilaria Stanzione, Rossana Pitocchi, Anna Pennacchio, Paola Cicatiello, Alessandra Piscitelli, Paola Giardina

**Affiliations:** Department of Chemical Sciences, University of Naples Federico II, Naples, Italy

**Keywords:** biosensors, functional amyloid, fusion proteins, coating, protein immobilization

## Abstract

Research on innovative surface functionalization strategies to develop materials with high added value is particularly challenging since this process is a crucial step in a wide range of fields (i.e., biomedical, biosensing, and food packaging). Up to now, the main applied derivatization methods require hazardous and poorly biocompatible reagents, harsh conditions of temperature and pressure, and are time consuming and cost effective. The discovery of biomolecules able to adhere by non-covalent bonds on several surfaces paves the way for their employment as a replacement of chemical processes. A simple, fast, and environment-friendly method of achieving modification of chemically inert surfaces is offered by hydrophobins, small amphiphilic proteins produced by filamentous fungi. Due to their structural characteristics, they form stable protein layers at interfaces, serving as anchoring points that can strongly bind molecules of interest. In addition, genetic engineering techniques allow the production of hydrophobins fused to a wide spectrum of relevant proteins, providing further benefits in term of time and ease of the process. In fact, it is possible to bio-functionalize materials by simply dip-casting, or by direct deposition, rendering them exploitable, for example, in the development of biomedical and biosensing platforms.

## Introduction

The process of surface functionalization plays a pivotal role in a wide range of application fields (i.e., medical, sensing, and food packaging), affecting the quantitative and qualitative aspects of several methodologies and products. That is why the research for innovative functionalization strategies is particularly challenging. Different strategies have been studied and optimized according to the nature of the surface to derivatize and the target application. There are plenty of examples in the literature, ranging from physical techniques (i.e., laser ablation, abrasive blasting, evaporation, and ion-assisted deposition) (Peng et al., 2016; Yang et al., 2017; Letzel et al., 2019; Wolf et al., 2021) to chemical treatments (i.e., chemical vapor deposition, plasma-assisted surface oxidation, nitration, hydrolyzation, and amination) (Javanbakht et al., 2015; Boaretti et al., 2020), that are commonly used. The mentioned strategies are affected by high costs and environmental impacts due to the inclusion of several time-consuming steps and often using hazardous reagents/solvents under harsh environmental conditions (Lavilla et al., 2014). In the case of biomolecules immobilization, other factors need to be considered such as good orientation of the active sites and retention of biological activity. Nonetheless, immobilized biomolecules (i.e., DNA, enzymes, and antibody) increase their stability against environmental changes and can be easily recycled (Ashkan et al., 2021). Starting from these considerations, the introduction of biocompatible and easy functionalization methodologies is taking place in different working sectors to replace standard methods. The discovery of biomolecules that can adhere to several surfaces with non-covalent bonds paves the way for their employment in these processes. A simple, fast, and environment-friendly means of achieving the modification of chemically inert surfaces is offered by the amphiphilic proteins, produced by filamentous fungi, known as hydrophobins (HPBs) (Fan et al., 2015). HPBs are small cysteine-rich proteins, ranging from 70 to 350 amino acids, expressed at different stages of the fungal life cycle, fulfilling multiple functions in fungal development. These proteins self-assemble at hydrophobic/hydrophilic interfaces into amphipathic layers, changing the wettability of surfaces and playing a role in adhesion and surface modification, acting as coating/protective agents (Cicatiello et al., 2020). It is common knowledge that HPBs can be divided into two classes that correspond to class I HPBs, self-assembling into resistant layers similar to amyloid fibrils that can be solubilized only by means of strong acids, and class II HPBs that form less regular and stable assemblies soluble in solvents and detergents (Ball et al., 2020). Adhesion of HPBs is a very fast process; indeed, they can tightly bind several surfaces by dip-casting or direct deposition. Using an engineered Class II HPB variant (Szilvay et al., 2006) linked to the atomic force microscopy cantilever tip, Paananen et al. demonstrated that interactions between HPB and hydrophobic surfaces are stronger than those formed between two hydrophobin molecules (Paananen et al., 2021). Lo et al. demonstrated that Class I HPB layers formed on a graphite surface are resistant to alcohol, acid, and basic washes (Lo et al., 2014). In contrast, a Class II monolayer is dissociated by alcohol treatment, even if relatively stable towards acid and base washes. However, it is worth noting that HPBs can exhibit various surface binding characteristics, even if they belonged to the same class and to the same organism (Winandy et al., 2018).

These characteristics, together with the capability of the HPB layer to immobilize a plethora of biomolecules by adsorption, make them appealing candidates as biological linkers, exploitable across a wide range of applications. For example, HPB on a polymeric surface can function as an “ink receiving layer,” rendering the surface amenable to ink jet printing with desired features (Misra et al., 2015).

More recently, a smart strategy is offered by the production of HPB fusion proteins, obtained using genetic engineering techniques to couple the adhesive capability of HPBs with the biological activity of different target proteins, leading to a rapid and efficient immobilization process (Kurppa et al., 2014; Hennig et al., 2016).

**FIGURE 1:**
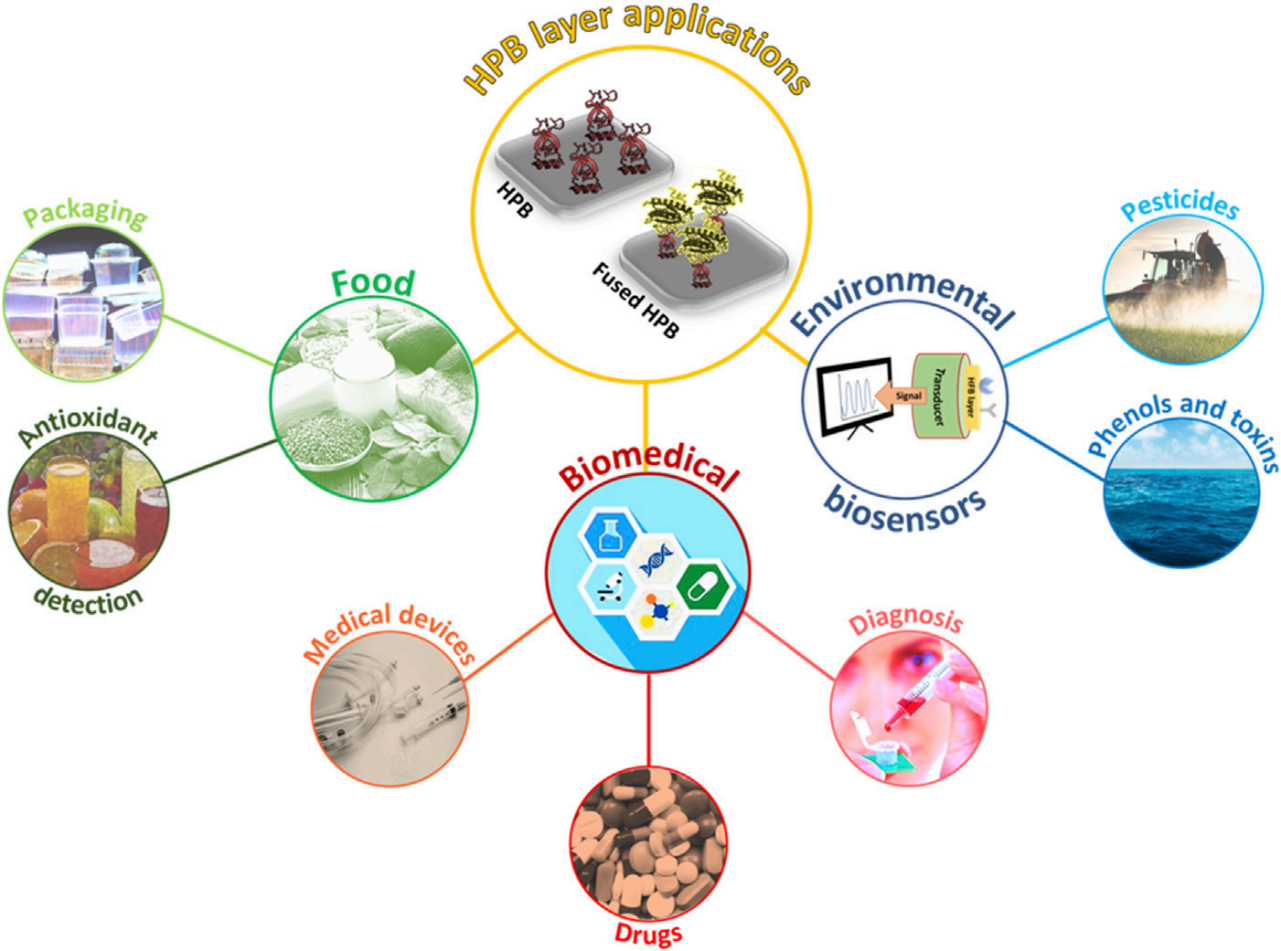
Schematic representation of HPB layer exploitation in different application fields.

In this work, we provide an overview of the most recent (last ten years) exploitations of wild type and fused HPBs in the development of innovative biosystems in food, environmental, and biomedical fields (Figure 1).

## Environmental applications

Biosensors are considered a cutting-edge frontier in environmental diagnostics representing a rapid and cost-effective strategy, alternative to traditional methods (Scognamiglio et al., 2014), for real-time *in situ* analysis. One of the limiting steps in biosensor designing is represented by immobilization of bioreceptors on the transducer elements. The HPBs’ ability to bind several biomolecules in their active forms allowed the development of very versatile systems for the detection of a wide range of environmental pollutants by changing the immobilized bioreceptor. For example, the HPB layers were used by Tao et al. to easily functionalize film bulk acoustic wave resonators (FBARs) enabling the development of a sensitive system with the ability to detect volatile organic compounds (VOCs) (Tao et al., 2019). VOCs represent biomarkers of several disorders and diseases (Fitzgerald et al., 2017); thus, their detection and quantification, traditionally carried out by chromatographic strategies, are very useful. The coating obtained by simple droplet casting of the HPB HFBI from *Trichoderma reesei* not only allowed a better adsorption of the analytes, but also their discrimination according to their polarity, due to the negatively charged nature of the HFBI film.

Regarding the exploitation of HPB fusion proteins, Piscitelli et al. (2017b) developed a glutathione-S-transferase (GST)–based biosensor to detect pesticides, such as molinate and captan, in aqueous environmental samples. The authors recombinantly produced in *Escherichia coli* the fusion protein composed by GST and the Class I HPB Vmh2 from *Pleurotus ostreatus*. Vmh2–GST fusion protein can be easily and rapidly immobilized on the polystyrene surface by direct deposition and is able to sense with high sensitivity and accuracy both toxic compounds, which act as competitors of an enzyme substrate. With a similar aim, Doring et al. (2019) fused the target protein 5-enolpyruvylshikimate-3-phosphate synthase (EPSPS) to the HPB Ccg2 (also known as EAS from *Neurospora crassa*) to functionalize both glass and polystyrene surfaces. Using this biosystem, the widespread herbicide glyphosate (Kanissery et al., 2019), which inhibits the enzyme activity (Murtaza and William, 2001), was detected at a nanomolar sensitivity. The surfaces were also functionalized with a mixture of the fusion protein and the wild type HPB to avoid steric hindrance, achieving better signal-to-noise (S/N) ratio. The same fusion protein was later used by Rettke et al. (2020) to functionalize a glass slide by direct deposition, thus developing an ultrasensitive optical particle-based biosensor. A straightforward approach to quantify the analyte was set up as a competitive binding assay between glyphosate immobilized on soft colloidal probe and the free one.

Among the well-known environmental pollutants, heavy metals are highly toxic, persistent, and capable of bioaccumulation in nature (Ali et al., 2019). Quantification of mercury, arsenic, cadmium, and nickel in aqueous samples is currently performed using elaborate methods. Biosensors based on the Vmh2 fusion proteins were set up as alternative methods for the detection of heavy metals. Puopolo et al. coupled the HPB Vmh2 with a thermophilic arsenate reductase, able to reduce As(V) to As(III) (Puopolo et al., 2021). The functionalized gold electrodes were used by the authors as innovative platforms for the detection of arsenic. Moreover, Pennacchio et al. (2022) developed a fluorescence-based biosensor based on Vmh2 fused to a mercury-binding peptide. The immobilized fusion protein was able to bind to mercury in the presence of other metals, leading to a decrease in the fluorescence intensity and achieving a nanomolar sensitivity both in tap and seawater. The real innovation of this biosensor is related to the combination of the fluorescence detection with machine learning methodologies to predict the mercury (II) concentration by analyzing the image of a multiwell plate, without the need of classical reader devices.

Stanzione et al. (2021) designed and expressed in *E. coli* two Vmh2 fusion proteins in which the HPB partners were single-chain fragment variables (ScFvs) of antibodies, able to recognize two marine neurotoxins, saxitoxin and domoic acid. These algal toxins are associated with algal blooms and can be bioaccumulated in fish or shellfish, leading to heavy consequences for human health (Vilariño et al., 2009). By immobilizing the proteins on pristine magnetic nanoparticles, both optical and electrochemical biosensors, able to detect the two neurotoxins, were developed.

Highly sensitive detection of catechol and poly aromatic hydrocarbons (PAH), widespread contaminants of the environment, was progressively developed starting from the fusion of Vmh2 with a laccase enzyme. Laccase is able to oxidize aromatic and phenolic compounds by reducing molecular oxygen to water. In this case, an added value of the system was the enzyme immobilization on carbon nanomaterials, thereby endowed with a high surface-to-volume ratio. Starting from previous experimental works that demonstrated the successful functionalization of nanomaterials with Vmh2 (Gravagnuolo et al., 2015), Sorrentino et al. (2020b) produced few-layer graphene functionalized with the Vmh2–laccase fusion protein through a “one-pot” approach. Once the immobilization conditions were optimized, biofunctionalized few-layer graphene was deposited on glass carbon electrodes to detect catechol at micromolar sensitivity. An improvement of this biosensor was later developed by immobilizing the same fusion protein on multi-walled carbon nanotubes reaching higher sensitivity (Sorrentino et al., 2021). More recently, Vmh2–laccase fusion protein was also immobilized on magnetic beads and used to detect three PAHs, upon their oxidation into quinones in the presence of a redox mediator. Owing to the efficient adsorption of quinones at carbon nanotube–modified electrodes, PAHs were detected at picomolar sensitivity (Sorrentino et al., 2022).

## Biomedical applications

HPBs are widely used for the formulation and the administration of hydrophobic drugs, for the development of biomedical devices, and for the detection of targeted biomarkers in diagnostics. Although many examples regarding the use of HPBs as effective adjuvants for improved hydrophobic drug solubility, stability, and bioavailability are reported (Weiszhár et al., 2012; Valo et al., 2013; Fang et al., 2014; Paukkonen et al., 2017), in this review, we will focus on HPB exploitation in the functionalization of drug carrier surfaces. Class II HPBs were used to functionalize silicon, gold, and other carriers, providing several benefits in terms of drug stability, biodistribution, and targeting. The coating of porous silicon with the HPB HFBII modified the hydrophobicity of the nanoparticles, improved intravenous biodistribution with respect to non-functionalized particles, and altered the adsorption profile of plasma proteins on the particle surface (Sarparanta et al., 2012). Maiolo et al. (2017) functionalized dodecanethyl-protected gold nanoparticles with the same HPB, obtaining exceptionally stable encapsulated drugs, whose release was selectively triggered in the tissues by the cytoplasmic glutathione reduction. More recently, Barani and coworkers coated the surface of doxorubicin-loaded niosomes with HFB-1, a vesicular system made from nonionic surfactants, presenting a promising drug delivery system for sustained drug release in cancer (Barani et al., 2020). Using a different approach, Reuter et al. (2017) improved drug delivery to specific target cells by functionalizing porous silicon nanoparticles with a fusion protein combining the coating ability of the HPB HFBIV with the targeting ability of the human transferrin. The nanoparticles were stable, degradable, non-immunogenic, and specifically reached their targets. The HPB–transferrin coating also affected the biodistribution of the nanoparticles when administered intravenously to rats and allowed decrease in the adsorption of plasma proteins around the nanoparticles and, consequently, their aggregation and loss of activity (Reuter et al., 2017). Furthermore, the Class I HPB HGFI from *Grifola frondosa* and its mutant were used to easily modify halloysite clay nanotubes loaded with an anticancer drug: results confirmed that the HPB coating enhanced the dispersion stability, the pH-dependent prolonging drug release, and the effective cytotoxicity (Wang et al., 2021).

Despite many breakthroughs in science and technology, a significant proportion of medical implants are still infected by bacteria. Implant infection is estimated to cause around one-fifth of implant failures, which can occur at any time post-implantation (VanEpps and Younger, 2016). Surface contamination represents a serious concern both in material science and in medicine; therefore, prevention and control of nonspecific protein adsorption and of microbial growth on surfaces is a key issue, currently addressed by many researchers (Wang et al., 2017). As the cell number keeps increasing on a surface, microbes usually start to build up a biofilm, namely, a complex structure consisting of polysaccharides, proteins, and extracellular DNA in which cells are embedded. The biofilm is characterized by a strong resistance to many treatments and consequently, is extremely hard to remove (Maan et al., 2020). Several approaches to prevent biofilm formation on implants include applying chemical surface coatings that kill bacteria such as antibiotics, antimicrobial peptides, and metal nanoparticles (Maan et al., 2020). The ability of Class I HPBs to reduce the thickness of the biofilm formed by different strains of *Staphylococcus epidermidis* on plastic-, metal-, and glass-coated surfaces has been demonstrated first by Artini et al. with confocal laser scanning microscope analysis (Artini et al., 2017). The HPB SC3 was used to coat a medical-grade nitric oxide (NO)–releasing polymer, providing an antifouling layer which works synergistically with NO’s antibacterial and antiplatelet activities (Devine et al., 2019). Moreover, fusion protein containing HPB and antimicrobial peptides went one step further. Wang and coworkers fused the HPB HGFI to the antimicrobial peptide pediocin PA-1 and proved the ability of the fusion protein against two Gram-positive bacteria (Wang et al., 2017). Sorrentino et al. (2020a) developed a chimeric protein combining the assessed capabilities of the HPB Vmh2 with those of the human antimicrobial peptide LL37. The so-developed fusion protein enhances and enlarges the anti-biofilm activity against both Gram-positive and Gram-negative pathogenic bacteria, and also displays biocidal activity, since dead cells were found in the biofilm layer.

In other fields of research, HPBs were fused to different protein motifs, and the coating they formed was used for the development of biocompatible surfaces able to allow both adhesion of human cells and tissue regeneration (Huang et al., 2013; Zhao et al., 2016; Wang et al., 2017). Boeuf et al. (2012) used the Class I HPB from *Aspergillus nidulans* to functionalize orthopedic implant surfaces. Two HPB variants were produced, one with fibronectin receptor sites (RGD) and another one with the laminin domain (LG3) and showed continuous adhesion of human cells without increasing the adhesion of bacterial cells (Bouef et al., 2012). In the patent of Shufang et al. (2017), the inventors set up a method to prepare a novel bioactive protein-loaded stent material using the amphiphilic protein HFBI. The stent material improves its biocompatibility and promotes cell migration and vascularization.

As far as medical diagnostics are concerned, different HPB functionalization strategies were adopted. Functionalization of optical fibers was achieved by exploiting the self-assembly of HPB and the immobilization of goat-anti-rabbit IgG for the development of a label-free immunosensor. HGFI allows the formation of a self-assembled amphipathic film on the fiber surface, which can adsorb antibodies with desirable advantages such as ease of use, quick response, good stability, good repeatability, good specificity, and no side effects (Duan et al., 2020). Following a different approach, the class II HPB HFBI was fused to Protein A, a protein known to bind different immunoglobulin subclasses, and the HFBI–Protein A monolayer was proven to bind to immunoglobin; thus, a highly sensitive label-free graphene biosensor was built (Soikkeli et al., 2016). Moreover, a Vmh2–fusion protein was used to detect thrombin in human plasma (Piscitelli et al., 2017a). In this case, the HPB was fused to the green fluorescent protein (GFP) and immobilized on multiwell plates. Since the two proteins are linked by the specific cleavage site of thrombin, the higher the protease concentration, the lower the fluorescence intensity measured in the well, due to the release of GFP from the surface, thus developing an ultrasensitive biosensor. A very similar approach was used by Zhang and coworkers by using a HGFI-fusion protein (Zhang et al., 2020). Mirzaei et al. coated glassy carbon electrodes with HFBI and used it as a glue to immobilize the lactate dehydrogenase enzyme developing a biosensor for pyruvate, whose concentration is an index of circulatory disorders (Mirzaei et al., 2019). The HPB layer improved enzyme stability and allowed the achievement of low detection limits. In the recent invention of a CRISPR high-throughput biochip for detecting single gene mutation, an HPB is used for modifying a chip substrate (Zefang et al., 2019). Compared with a common substrate, this chip allows a uniform spread of the protein on the surface, avoiding the waste of biomolecules.

## Food applications

The exploitation of HPBs in food industry is mainly related to their ability to stabilize emulsions, for instance, in the design of aerated foods (e.g., mayonnaise, shelf-stable milk shakes, smoothies and other beverages, yoghurt, and gelatin-free mousse), with benefits such as fat/calorie reduction or improved/new product textures. With regard to their capability to functionalize surfaces, HPBs were recently exploited to enable a biosensor to detect antioxidant compounds in beverages. These compounds are used to maintain the color stability and to protect beverages from oxidative deterioration. Nonetheless, high concentration of these can have negative effects on human health. Thus, to overcome the traditional chromatographic methods often used for their analysis, Sorrentino et al. (2019) developed an innovative, easy-to-use, and rapid colorimetric biosensor, immobilizing the already mentioned Vmh2–laccase on the polystyrene surface. A competitive assay between a chromogenic substrate of the laccase enzyme and the caffeic acid as inhibitor allowed reaching micromolar sensitivity in real samples, such as ACE juice and tea infusion.

A completely different application of HPBs was developed to improve the degradation of plastic materials used in food packaging. Polyethylene terephthalate (PET) is the most widely used plastic for beverage bottles and food packaging, although its low level of degradation has a heavy impact on the environmental pollution. Puspitasari et al. (2021) discovered that an HPB layer on a PET surface, not only changed its wettability, making the plastic component more hydrophilic, but also made its degradation by PETase enzyme easier. Thus, the HPB RoIA, extracted from the fungus *Aspergillus oryzae*, was successfully immobilized on the PET surface, obtaining faster and enhanced activity of the PETase enzyme when compared to its performance without the HPB layer. Moreover, a comparison between the effects of three free HPBs and of their fusion proteins with the cutinase enzyme was studied by Ribitsch et al. (2015) based both on the ability of cutinase enzyme to hydrolyze PET and of HPB to enhance its degradation. The authors demonstrated that the genetic fusion between the HPBs and the cutinase leads to a better performance of the enzyme. The HPB role should be the creation of a more hydrophilic surface, leading to a better binding and targeting of the cutinases to PET.

## Protein structure analysis and proteomic applications

**FIGURE 2:**
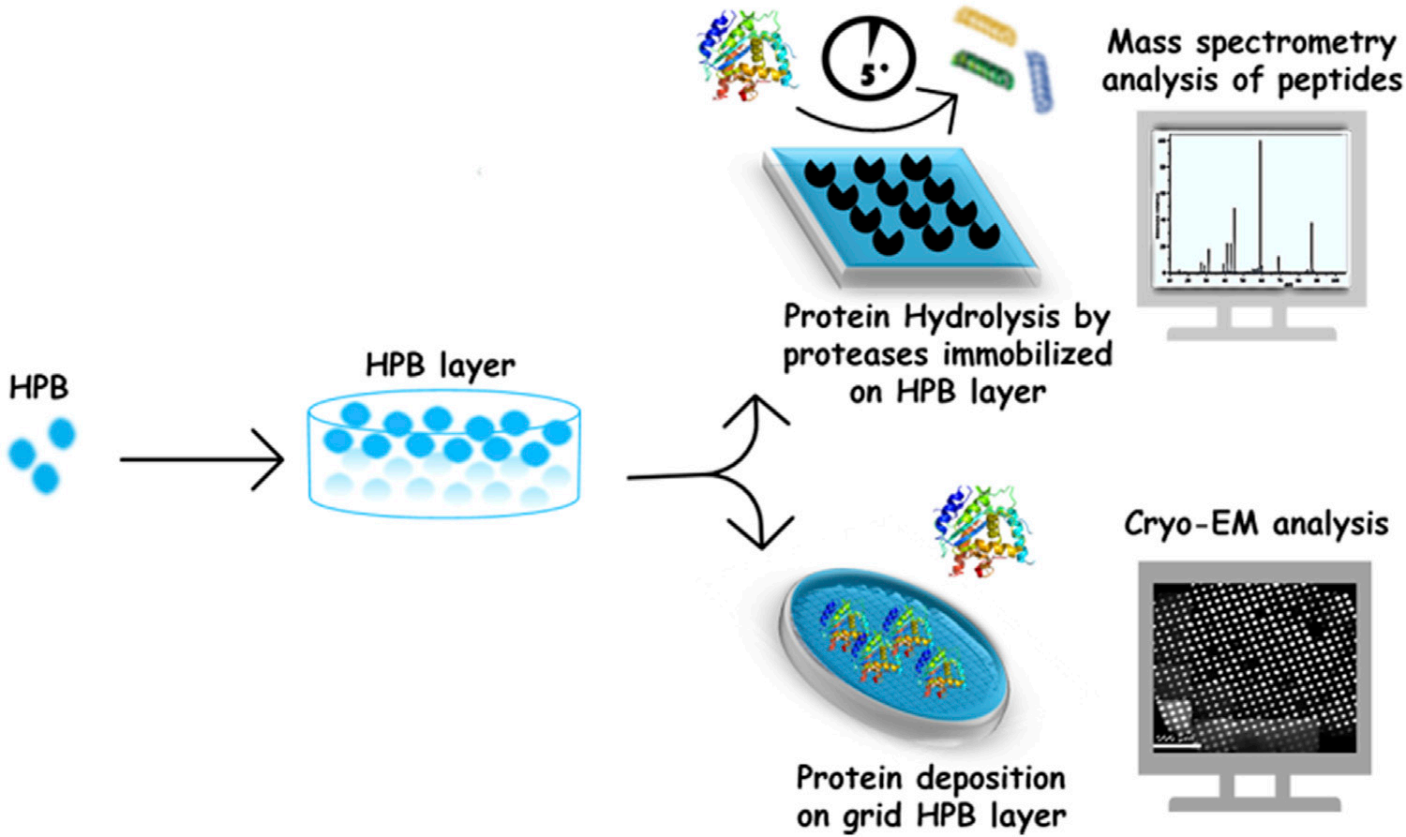
HPB layer used in protein structure analysis and proteomic applications.

The HPB layers were successfully exploited to improve the adhesion of proteins on supports used in different structural analysis techniques (Figure 2).

Using the class II HPB HFBI, a cryo-EM support film was developed. The HFBI film, spontaneously formed on top of a solution drop, was transferred onto the cryo-EM grid, with a simple and highly reproducible procedure, avoiding the need for conventional grid treatments before cryo-vitrification. The hydrophilic side of HFBI film adsorbs proteins via electrostatic interactions, allowing high-quality data collection. Using this support, the authors determined high resolution structures of some proteins (e.g., aldolase 150 kDa, hemoglobin 64 kDa), demonstrating the potentiality of HFBI films in increasing the success rate and efficiency of cryo-EM analyses (Fan et al., 2021).

Regarding mass spectrometry analysis, the self-assembled layer of the Class I HPB Vmh2 was successfully used to functionalize the conductive supports used in direct matrix-assisted laser desorption/ionization (MALDI) mass spectrometry. Indeed, the Vmh2 coating enables an efficient on-plate desalting and, consequently, a fast analysis with a good S/N ratio of a wide mass range of molecules (from small peptides to intact proteins), thus representing a very simple alternative to other known methods (Longobardi et al., 2014). In a different scenario, Vmh2-coated MALDI sample plates were exploited as a lab-on-plate platform for a stable non-covalent and non-destructive immobilization of enzymes commonly involved in proteomic studies (such as trypsin, V8 protease, PNGaseF, and alkaline phosphatase). Efficient, fast, and reproducible protein digestion is of utmost importance in bottom-up proteomics. Immobilization of proteolytic enzymes has gained an increasing interest owing to its several advantages over soluble enzyme reactions. Most importantly, it allows online mass spectrometric (MS) analysis of the proteolytic digests and effectively suppresses autoproteolysis even at high enzyme-to-substrate ratios (Tähkä et al., 2019). Rapid and efficient on-plate reactions were performed on Vmh2-coated MALDI plates, using very low sample volumes (some microliters) achieving high protein sequence coverage in few minutes, even when multi-proteolysis steps were performed directly on the same plate (Longobardi et al., 2015). This approach was exploited for *in situ* detection on a MALDI plate of blood as a real sample. In particular, the authors demonstrated the opportunity to discriminate the blood provenance even when present in a mixture (Patel et al., 2016).

In the field of cultural heritage preservation, the self-assembled Vmh2 layer was employed to bio-functionalize a flexible cellulose acetate sheet with trypsin, to digest proteins and capture peptides from paintings and ancient archeological artifacts with good efficiency. This flexible tool was fully portable without the need of microsampling the works of art; indeed, it is only necessary to moisten the biofunctionalized sheet just before use, and put it in contact with the work of art. The released peptides are captured onto the film surface and then analyzed when required, by a simple MALDI-TOF analysis or, eventually, by more resolutive and informative LC–MS/MS analyses (Cicatiello et al., 2018). Additionally, this lab-on-plate method was implemented immobilizing the glycosidases PNGaseF on the Vmh2-coated sheet for a two-step procedure in which the deglycosylation pretreatment allows an improved proteolysis and detection of glycosylated proteins (Ntasi et al., 2021).

Because of HPBs’ natural abilities, these interesting proteins can be used to replace conventional strategies generally adopted in surface derivatization processes. Nonetheless, some weak points of HPB exploitation have to be considered, for example, the unpredictability of the ligand–HPB layer interactions and the low production yield of native HPBs, which hinder their employment at industrial scale. Therefore, the recombinant production of HPBs, wild type or fused to target proteins, can allow researchers to tackle these issues. However, this strategy still carries some problems since it is not feasible for non-protein ligands and production of functional HPB fusion proteins cannot be assumed *a priori.*

Further studies on the potential of HPBs can encourage their use, even in unexplored fields of application, making them building blocks for macroscopic functional materials.
